# Improving the Use of Medicines Reconciliation Information in Inpatient Prescribing on an Elderly Care Ward: A Quality Improvement Project

**DOI:** 10.7759/cureus.117026

**Published:** 2026-09-27

**Authors:** Kogul Kamalanathan

**Affiliations:** 1 Internal Medicine, Hampshire Hospitals NHS Foundation Trust, Winchester, GBR

**Keywords:** electronic prescribing, medication discrepancies, medication reconciliation, medicines reconciliation, older adults, patient safety, prescribing safety, quality improvement

## Abstract

Background

Medicines reconciliation (MR) provides an accurate list of a patient’s medications, but its clinical utility depends on the reconciled list being accurately translated into the inpatient prescription and intentional changes being documented. Older patients are particularly vulnerable because of polypharmacy, multimorbidity, and frequent transitions between care settings.

Objective

The primary objective of this study is to assess regular medication prescribing against the completed MR list after transfer to an elderly care ward, with a target of a 50% relative reduction in patients with at least one clinically important unexplained discrepancy. A secondary objective was to assess the documented rationale for intentional medication changes.

Materials and methods

A retrospective quality improvement project was conducted at a district general hospital in England. During the baseline period (May-July 2025), 75 patient records were reviewed, of which 71 were included after excluding four without an MR list. Inpatient regular medication prescriptions were compared with the MR list, and the electronic patient record was reviewed for documented rationale. Discrepancies with a documented rationale were classified as intentional and were not counted as unexplained discrepancies. Minor discrepancies involving low-consequence medications were excluded from the primary analysis. The intervention consisted of a resident doctor induction briefing on accessing and using the MR list and a workflow reminder poster in the doctors’ room. A further 75 records were reviewed from September-November 2025, with 71 included after the same exclusions. Fisher’s exact test and risk ratios with 95% confidence intervals (CIs) were calculated.

Results

Among the 71 patients analysed in each period, the proportion with at least one unexplained discrepancy fell from 21 (29.6%) to 15 (21.1%), a relative reduction of 28.6% (risk ratio 0.71, 95% CI 0.40-1.27; p=0.335). Among patients with clinically important discrepancies, the proportion for whom every discrepancy had documented rationale increased from 9 (30.0%) of 30 patients to 24 (61.5%) of 39 patients (risk ratio 2.05, 95% CI 1.13-3.74; p=0.015).

Conclusion

A low-cost educational and visual reminder intervention was associated with fewer unexplained discrepancies in regular medications and substantially better documentation of intentional medication changes. The reduction in unexplained discrepancies was not statistically significant and the pre-specified target of a 50% relative reduction was not achieved. Further cycles will aim to strengthen pharmacist-doctor communication and assess the severity of discrepancies.

## Introduction

Accurate regular medication prescribing is paramount in maintaining patient safety. The World Health Organization’s Medication Without Harm challenge aimed to reduce severe avoidable medication-related harm by 50%, with transitions of care identified as one of three key action areas [1]. The National Institute for Health and Care Excellence (NICE) recommends that, in acute settings, medicines reconciliation (MR) should be completed within 24 hours of admission or sooner when clinically necessary [2,3].

MR involves comparing the best available medication history with what is currently prescribed to identify any discrepancies. Admission discrepancies are common; in a prospective study of medical patients taking at least four regular medications, 81 (53.6%) of 151 patients had at least one unintended discrepancy and omission was the most frequent error [4]. However, a reconciled list does not guarantee accurate prescribing of regular medications. The list still needs to be translated by clinicians into the inpatient prescription. In addition, if the rationale for an intentional medication change is not documented, subsequent clinicians may have difficulty distinguishing an appropriate clinical decision from an omission.

Older inpatients are particularly vulnerable to harm from medication discrepancies because polypharmacy, multimorbidity, frailty, cognitive impairment, and repeated transfers increase the complexity of medication decisions. Prescribing errors are also multi-factorial; a qualitative study with resident doctors identified knowledge gaps, workload, routines, communication, and supervision as contributing factors [5].

At our hospital, patients transferred to an elderly care ward generally had an MR list already completed and available within the electronic patient record. The critical step depended on the resident doctors using this list, comparing it with the Electronic Prescribing and Medicines Administration (EPMA) prescription, and reviewing any unexplained discrepancies. The baseline audit identified frequent unexplained discrepancies. This suggested that, despite an MR list being available, discrepancies between the list and the inpatient prescription were not consistently resolved or explained following ward transfer.

The Medications at Transitions and Clinical Handoffs (MATCH) Toolkit for Medication Reconciliation recommends that an MR list should be clearly identifiable and incorporated into staff training and the routine clinical workflow [6]. A UK-based quality improvement study found that providing resident doctors with access to electronic pre-admission medication information, together with targeted teaching, improved the completeness of MR [7]. In another paper involving an American hospital, clinician education, outreach, and an electronic reminder increased clinicians’ use of an existing electronic MR tool from 198 (18%) of 1,114 eligible patients to 155 (41%) of 376 eligible patients [8]. Although these interventions were implemented at different stages of the MR process, they support the rationale that clinicians are more likely to use medication information already present in the electronic record when they understand how to access it and are reminded to do so within their normal workflow.

Our intervention was therefore implemented to address unfamiliarity with EPMA and subsequent failure to routinely access the completed MR list. The intervention was a brief induction session demonstrating to resident doctors how to locate the MR record, compare it with the inpatient prescription, and amend or document identified discrepancies. In addition, a poster was displayed in the doctors’ office to reinforce this process throughout the working day. 

This quality improvement project aimed to assess the accuracy of regular medication prescribing following transfer to an elderly care ward, rather than the completion of medicines reconciliation itself. The improvement target was a 50% relative reduction in the proportion of patients with at least one clinically important unexplained discrepancy following the intervention. The secondary objective was to assess the proportion of patients with clinically important discrepancies for whom every discrepancy had documented rationale.

This project was previously presented as an e-poster at the Emergency Physician’s International Conference (EPIC26) on July 9, 2026.

## Materials and methods

Setting and study design

This was a retrospective, single-centre quality improvement project undertaken on an elderly care ward at Royal Hampshire County Hospital, England. It consisted of an initial baseline audit, one intervention cycle, and a post-intervention audit. The manuscript was structured with reference to the SQUIRE 2.0 reporting guidelines [9].

The baseline audit covered May, June, and July 2025. A total of 75 patient records were reviewed, with a sample of 25 records per month. The post-intervention cycle covered September, October, and November 2025 and used the same sample size of 25 patient records per month. For each month, a starting date around the middle of the month was selected according to reviewer availability, and the next 25 consecutive ward transfers identified from the electronic ward transfer list were reviewed in chronological order. Records without an MR list were excluded after selection and were not replaced.

Patients were eligible if they had transferred to the ward during the relevant audit period and had an inpatient electronic prescription with an MR list available for comparison. Records without an MR list were excluded from the analysis because no reference list was available against which prescribing accuracy could be assessed.

Data collection and definitions

For this project, “regular medications” encompassed all ongoing community medications on the pre-admission medication list, including those which are pro re nata (PRN).

For each patient, the regular medications prescribed on the EPMA system were compared with the MR list. Discrepancies were categorised as omission, incorrect dose, incorrect frequency, incorrect timing, duplication, or incorrect medication. The electronic patient record was then reviewed for documented rationale. A discrepancy was treated as having documented rationale when a clinically relevant reason was documented in the electronic patient record, such as acute kidney injury, hypotension, bleeding, or adverse effects. An unexplained discrepancy was defined as a clinically important discrepancy for which no rationale was documented in the electronic patient record.

All regular medication discrepancies were reviewed. A discrepancy was considered clinically important if it had the potential to cause patient discomfort or clinical deterioration, taking into account the medication, indication, and clinical context. Discrepancies considered unlikely to have these effects were classified as minor and excluded from the primary outcome. Examples of discrepancies generally classified as minor included routine multivitamins without an indication, simple emollients, and lubricating eye drops. These were not automatically excluded if the patient’s clinical circumstances made their omission potentially important. Borderline cases were classified by the same reviewer using these criteria; no independent second review was undertaken.

A patient with one or more qualifying unexplained discrepancies was only counted once. Patients with both explained and unexplained discrepancies were classified as having an unexplained discrepancy. The proportion of patients with documented rationale for all clinically important discrepancies was also measured. One clinician reviewed all records and recorded the findings. The reviewer was aware of whether records were from the baseline or post-intervention period. Findings were recorded using the same structured data-collection spreadsheet in both periods.

Intervention

A fishbone diagram (Figure 1) was used to explore possible causes of discrepancies between the MR list and the inpatient prescription. 

**Figure 1:**
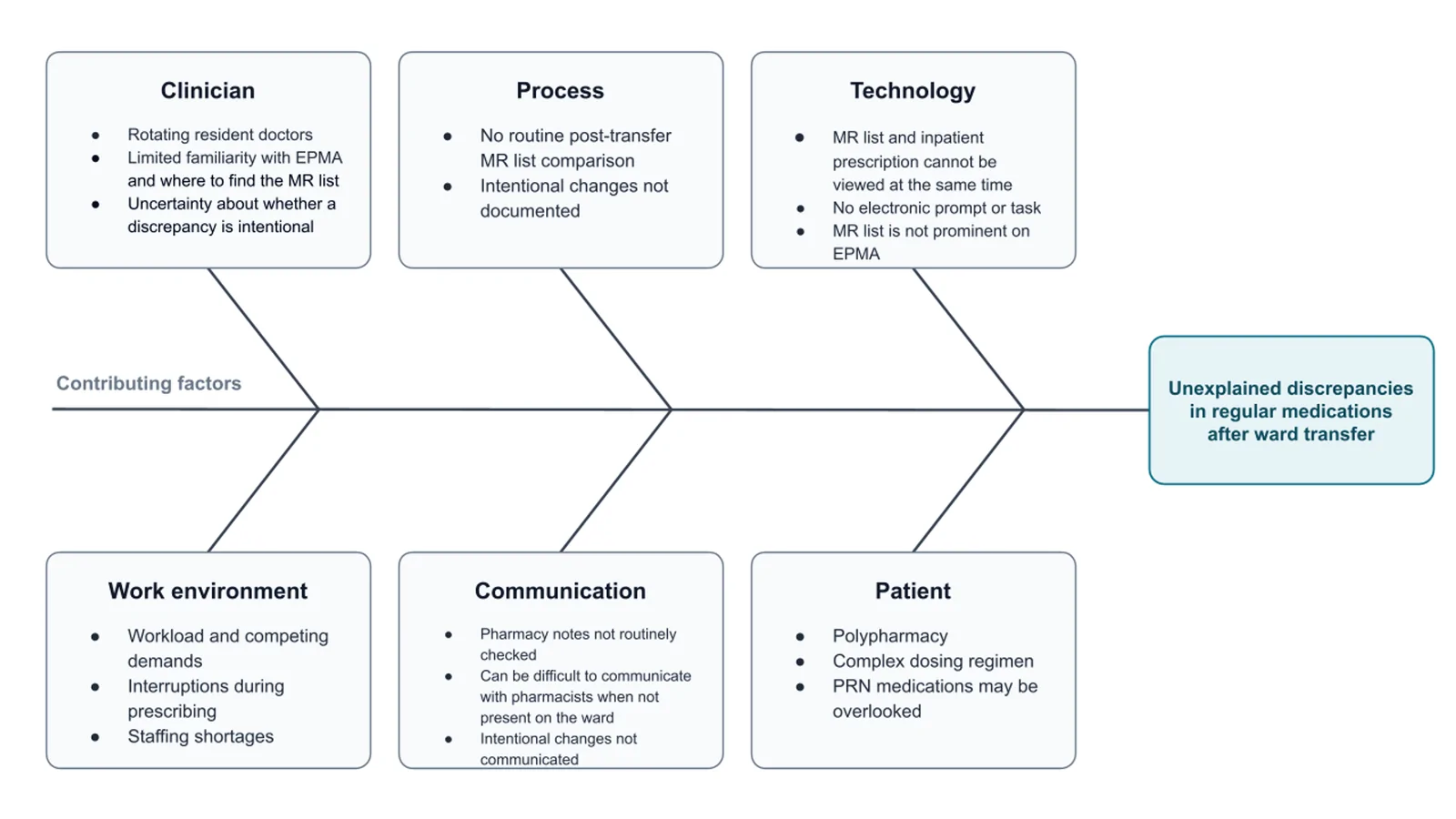
Fishbone diagram showing potential causes of discrepancies between the MR list and the inpatient prescription MR, medicines reconciliation; EPMA, electronic prescribing and medicines administration; PRN, pro re nata (as required).

Unfamiliarity with locating the list on EPMA was the main modifiable factor addressed in this cycle. The intervention consisted of two components that were introduced in August 2025. Five doctors worked on the ward during the post-intervention period.

First, the three doctors who were new to the Trust received a five-minute induction briefing from a senior house officer, explaining how to locate the MR list on EPMA and compare it with the inpatient prescription after a patient arrives on the ward. The other two doctors were already familiar with this process and did not receive the briefing. Records were included regardless of whether the prescribing doctor had attended the briefing.

Second, an informative poster was placed in the doctors’ room to reinforce this workflow and remind clinicians to document changes in regular medications. The poster remained in the room throughout the duration of the post-intervention cycle.

Outcome measures and statistical analysis

Primary Outcome Measure

The primary outcome measure is a proportion of patients with at least one unexplained discrepancy in regular medications.

Secondary Process Measure

The secondary process measure is a proportion of patients with clinically important discrepancies for whom every discrepancy had documented rationale.

Target

The target is a 50% relative reduction in the primary outcome compared with baseline.

Post-intervention proportions were compared with baseline using risk ratios and 95% confidence intervals (CIs) calculated with the log method. Baseline and post-intervention proportions were compared using separate two-sided Fisher’s exact tests. The same analyses were applied to the secondary process measure.

Ethical and governance considerations

This project was registered with Hampshire Hospitals NHS Foundation Trust as a quality improvement project. Only anonymised routinely collected clinical data were analysed, and no patient-identifiable information was recorded. The Health Research Authority decision tool classified the project as not being research; therefore, NHS Research Ethics Committee review was not required.

## Results

Study population

Across both periods, 150 records were reviewed. Four records in the baseline period and four in the post-intervention period were excluded because no MR list was available. The final analysis therefore included 71 patients in each period (142 patients overall).

Primary outcome

At baseline, 21 (29.6%) of 71 patients had at least one unexplained discrepancy, compared with 15 (21.1%) of 71 after the intervention. This was an absolute reduction of 8.5 percentage points and a relative reduction of 28.6% (risk ratio 0.71, 95% CI 0.40-1.27; Fisher’s exact p=0.335). Monthly results are shown in Figure 2.

**Figure 2:**
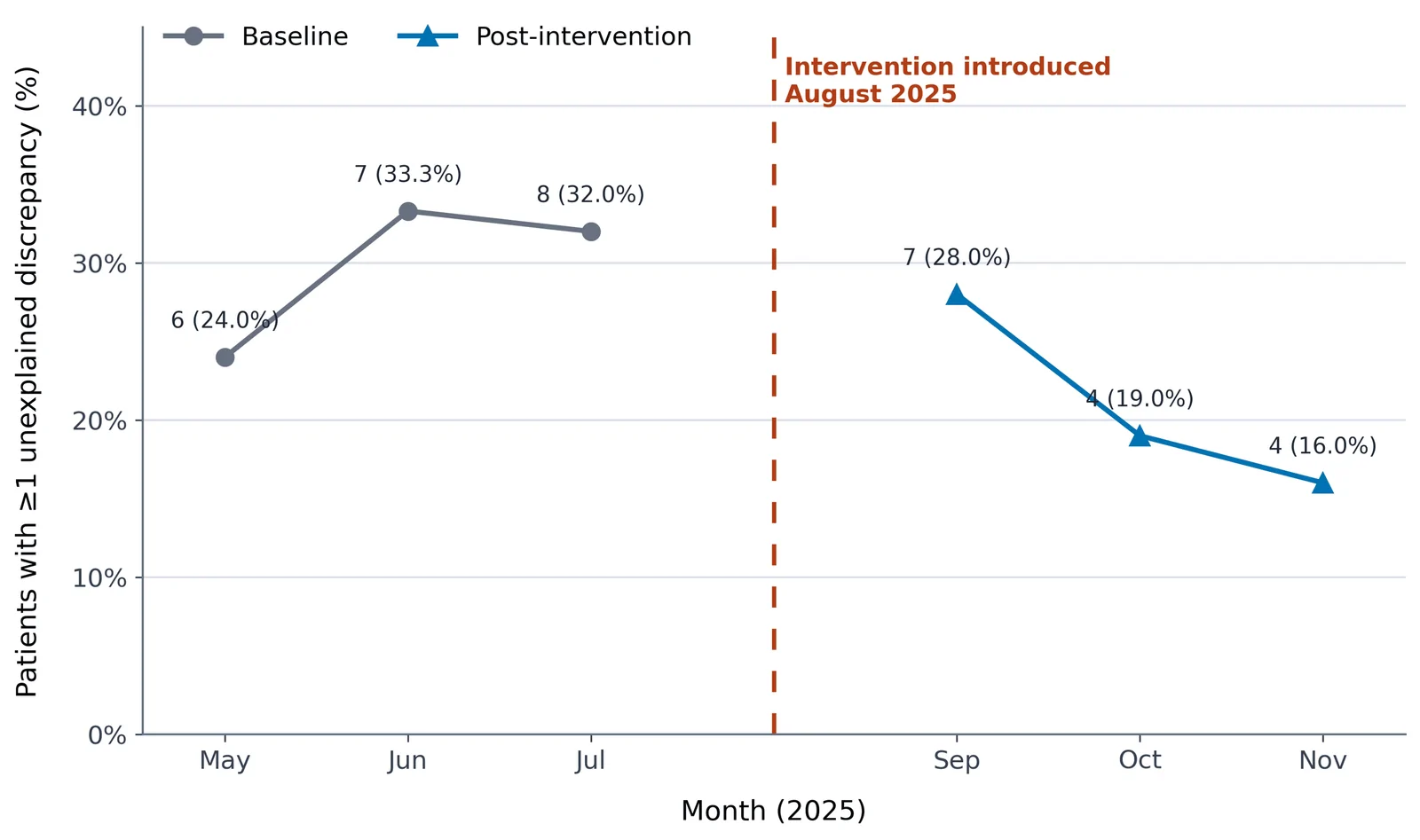
Monthly proportion of patients with at least one clinically important unexplained medication discrepancy Values are presented as N (%), where N is the number of patient records with at least one clinically important unexplained medication discrepancy. The monthly denominators were 25 patient records in May, 21 in June, 25 in July, 25 in September, 21 in October, and 25 in November. The dashed vertical line indicates introduction of the intervention in August 2025. Grey circles represent baseline data and blue triangles represent post-intervention data.

Secondary process measure

At baseline, 30 patient records had at least one clinically important discrepancy, compared with 39 after the intervention. All clinically important discrepancies had documented rationale in 9 (30%) of 30 patients at baseline and 24 (61.5%) of 39 patients after the intervention (risk ratio 2.05, 95% CI 1.13-3.74; Fisher’s exact p=0.015).

Baseline and post-intervention outcomes are summarised in Table 1.

**Table 1:** Medication discrepancy outcomes before and after the intervention Values are presented as N (%), where N is the number of patient records. For the primary outcome, the denominator was all patient records included in the analysis (N=71 in each period). For the secondary outcome, the denominator was patient records containing at least one clinically important discrepancy (baseline N=30; post-intervention N=39). CI, confidence interval.

Outcome	Baseline, N (%)	Post-intervention, N (%)	Risk ratio	95% CI	Fisher’s exact p-value
Primary outcome: patient records with at least one clinically important unexplained discrepancy	21 (29.6%)	15 (21.1%)	0.71	0.40–1.27	0.335
Secondary outcome: patient records in which all clinically important discrepancies had a documented rationale	9 (30.0%)	24 (61.5%)	2.05	1.13–3.74	0.015

## Discussion

Following a brief induction intervention and a visual reminder, the proportion of patients with at least one unexplained discrepancy in regular medications decreased from 21 (29.6%) to 15 (21.1%) out of 71 patients. This represented a 28.6% relative reduction, although the wide 95% confidence interval meant that a statistically significant difference was not demonstrated.

The clearest change was in the documentation of discrepancies. The proportion with documented rationale for all clinically important discrepancies increased from 9 (30.0%) of 30 patients to 24 (61.5%) of 39 patients. Clear documentation is important because medications are frequently withheld, adjusted, or appropriately substituted during acute illness; however, without documented rationale, other clinicians may restart a medication prematurely or continue an unintended omission that persists on discharge into the community. The secondary measure was restricted to patients with clinically important discrepancies. The number of patients in this group differed between periods, and unmeasured differences in case mix or the discrepancies themselves may have influenced the documentation rate.

The intervention did not achieve the initial target of a 50% relative reduction. This may be because it addressed only one part of a wider prescribing process. Resident doctor prescribing errors arise from many interacting individual and organisational factors, including practical knowledge, workload, interruptions, lack of supervision, and communication [5]. A short induction and poster may improve awareness but would not address these other factors.

Comparison with existing evidence

The baseline rate of unexplained discrepancies is consistent with literature showing frequent discrepancies at transitions of care. Cornish et al. found that over half of selected medical patients had an unintended admission discrepancy and that omission was the most frequent type [4]. Their study concerned admissions rather than ward transfers, but the underlying problem is similar: information needs to be carried across a change in environment and converted into accurate prescribing.

Most studies assess MR as a multi-component process, often combining medication history-taking, pharmacist input, and the correction of discrepancies [10,11]. Fewer have examined whether reconciled medication information is correctly reflected in inpatient prescriptions. Lee et al. found that 80 (62.0%) of 129 patients had at least one unintentional discrepancy between the best possible medication transfer list and the prescriptions written after transfer between wards [12]. Our project focused on this prescribing stage by encouraging doctors to compare the MR list with the inpatient prescription and to document the reasons for any intentional changes.

Educational interventions have been shown to reduce medication errors, although these interventions generally addressed prescribing knowledge rather than the use of the MR list [13,14]. Some combined teaching with written reminders or feedback [14]. We applied similar interventions to a different problem: familiarising doctors with the process of checking the MR list against the inpatient prescription.

A second cycle is currently underway, focusing on communication between pharmacists and doctors. For this cycle, a Microsoft Teams communication route was set up to allow pharmacists to flag unresolved medication discrepancies directly to the medical team rather than relying on pharmacy notes alone. This cycle will assess whether it leads to better documentation of intentional changes and fewer unexplained discrepancies. The original set of outcome measures will be repeated, with use of the Teams route recorded as an additional process measure.

Strengths

Our project examined a clinically important interface between MR and inpatient prescribing rather than measuring completion of reconciliation alone. It also attempted to distinguish intentional medication changes from unexplained discrepancies by reviewing the electronic patient record for documented rationale. This approach decreases the risk of classifying documented appropriate de-prescribing, substitution, or temporary suspension as an unexplained discrepancy.

The intervention itself was inexpensive, easy to deliver, and suitable for repetition when resident doctors rotate. The same data collection approach and sample size were used before and after the intervention, and the primary outcome was patient-based, allowing clinical teams to interpret the findings easily.

Limitations

This was a single-ward, uncontrolled before-and-after quality improvement project with a modest sample size, which limits the generalisability of the findings and means that changes observed post-intervention cannot be attributed solely to the intervention. Differences in staffing, patient mix, or pharmacy input between the two periods may also have influenced the results.

In addition, records were sampled around the middle of each month rather than across the full audit period, so they may not have been fully representative of all ward transfers. As there were only three monthly observations in each period, the apparent fall across the post-intervention months could reflect ordinary variation rather than a sustained improvement. 

Patients without an MR list were excluded because prescribing accuracy could not be assessed against a reference list; as a result, the project did not evaluate failures to complete MR itself. Patient characteristics, including frailty, number of regular medications, and discrepancy severity, were not collected. It was therefore not possible to assess whether the two groups were comparable in case mix or medication burden, or to determine the potential clinical impact of the discrepancies.

The classification of discrepancies depended on documentation in the electronic patient record and the clinical judgement of a single reviewer who was aware of the study periods. No validated severity framework or independent second review was used, so the distinction between clinically important and minor discrepancies was open to subjective interpretation. Some discrepancies categorised as unexplained may have represented appropriate clinical decisions for which the rationale had not been recorded. Adherence to the suggested workflow was not measured, so it is unclear how consistently doctors checked the MR list against the inpatient prescription.

Finally, minor discrepancies were excluded from the primary outcome, which focused the analysis on potentially more clinically important discrepancies, but this may reduce comparability with studies that report all medication discrepancies.

## Conclusions

A brief induction session and workflow reminder poster were associated with a reduction in unexplained discrepancies in regular medications following transfer to an elderly care ward and with a marked increase in documented rationale for intentional discrepancies. The reduction in unexplained discrepancies was not statistically significant and did not meet the target. A further quality improvement cycle is underway to improve pharmacist-doctor communication. Subsequent interventions could aim to embed MR review into the electronic workflow, measure the severity of discrepancies and assess intervention sustainability.

## Figures and Tables

**Figure 3:**
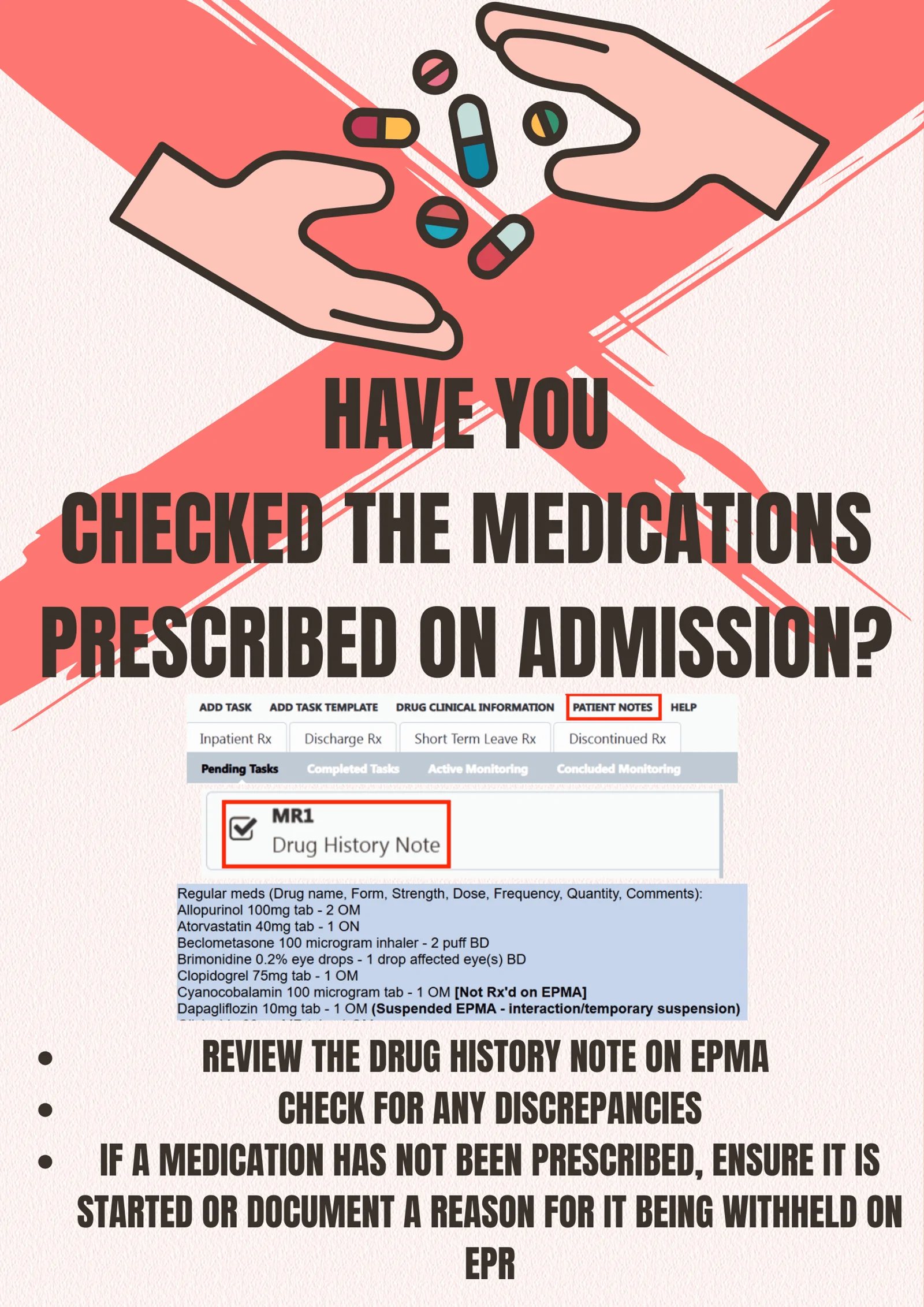
Reminder poster displayed in the doctors’ room to reinforce use of the medicines reconciliation list and to improve documentation of intentional medication changes. Poster created using Canva.com

## References

[1] (2026). Medication Without Harm: WHO Global Patient Safety Challenge on Medication Safety. https://www.who.int/publications/i/item/WHO-HIS-SDS-2017.6.

[2] (2026). Medicines optimisation: the safe and effective use of medicines to enable the best possible outcomes (NG5). https://www.nice.org.uk/guidance/ng5.

[3] (2026). Medicines optimisation (QS120): quality statement 4, medicines reconciliation in acute settings. https://www.nice.org.uk/guidance/qs120/chapter/quality-statement-4-medicines-reconciliation-in-acute-settings.

[4] Cornish PL, Knowles SR, Marchesano R, Tam V, Shadowitz S, Juurlink DN, Etchells EE (2005). Unintended medication discrepancies at the time of hospital admission. Arch Intern Med.

[5] Lewis PJ, Ashcroft DM, Dornan T, Taylor D, Wass V, Tully MP (2014). Exploring the causes of junior doctors' prescribing mistakes: a qualitative study. Br J Clin Pharmacol.

[6] Gleason KM, Brake H, Agramonte V, Perfetti C (2012). Medications at Transitions and Clinical Handoffs (MATCH) Toolkit for Medication Reconciliation. AHRQ Publication No. 11(12)-0059. Agency for Healthcare Research and Quality, Rockville, MD; revised August 2012.

[7] Iddles E, Williamson A, Bradley A, Khan K (2015). Are we meeting current standards in medicines reconciliation? A study in a district general hospital. BMJ Qual Improv Rep.

[8] Schnipper JL, Liang CL, Hamann C (2011). Development of a tool within the electronic medical record to facilitate medication reconciliation after hospital discharge. J Am Med Inform Assoc.

[9] Ogrinc G, Davies L, Goodman D, Batalden P, Davidoff F, Stevens D (2016). SQUIRE 2.0 (Standards for QUality Improvement Reporting Excellence): revised publication guidelines from a detailed consensus process. BMJ Qual Saf.

[10] Mueller SK, Sponsler KC, Kripalani S, Schnipper JL (2012). Hospital-based medication reconciliation practices: a systematic review. Arch Intern Med.

[11] Mekonnen AB, McLachlan AJ, Brien JA (2016). Effectiveness of pharmacist-led medication reconciliation programmes on clinical outcomes at hospital transitions: a systematic review and meta-analysis. BMJ Open.

[12] Lee JY, Leblanc K, Fernandes OA (2010). Medication reconciliation during internal hospital transfer and impact of computerized prescriber order entry. Ann Pharmacother.

[13] Ross S, Loke YK (2009). Do educational interventions improve prescribing by medical students and junior doctors? A systematic review. Br J Clin Pharmacol.

[14] Jaam M, Naseralallah LM, Hussain TA, Pawluk SA (2021). Pharmacist-led educational interventions provided to healthcare providers to reduce medication errors: a systematic review and meta-analysis. PLoS One.

